# RUFY3 promotes the progression of hepatocellular carcinoma through activating NF-κB-mediated epithelial-mesenchymal transition

**DOI:** 10.18632/aging.203444

**Published:** 2021-09-12

**Authors:** Yang Zhang, Weixing Ni, Lei Qin

**Affiliations:** 1 Department of General Surgery, The First Affiliated Hospital of Soochow University, Suzhou, Jiangsu Province, China; 2 Department of General Surgery, Yancheng City No.1 People’s Hospital, Yancheng, Jiangsu Province, China

**Keywords:** RUFY3, epithelial-mesenchymal transition, hepatocellular carcinoma, NF-κB

## Abstract

RUFY3 (RUN and FYVE domain-containing protein 3) has been demonstrated to exhibit carcinogenic effect in multiple malignancies. However, the exact role of RUFY3 in hepatocellular carcinoma (HCC) progression remains elusive. Herein, we aimed to identify the role and the underlying mechanism of RUFY3 in HCC progression. The RUFY3 levels in HCC specimens were detected by qRT-PCR, western blot, and immunohistochemistry, and its clinical significance in HCC patients was assessed. The effect of RUFY3 on HCC cell growth, migration, and invasion was explored by CCK-8 assay, wound healing assay, and transwell migration and invasion assays *in vitro*. The effect of RUFY3 on HCC cell growth and metastasis was also conducted *in vivo* through establishing xenograft tumor and lung metastatic mice model. The underlying mechanism responsible for RUFY3-induced HCC cell behavior was also investigated. Our results indicated that high levels of RUFY3 significantly correlated with tumor size, microvascular invasion, clinical stage, and poor prognosis for HCC patients. In addition, RUFY3 facilitated HCC cell growth, invasion, and metastasis both *in vitro* and *in vivo* through activating nuclear factor-κ-gene binding (NF-κB)-mediated epithelial-mesenchymal transition (EMT). Taken together, our results revealed that RUFY3 accelerated HCC progression via driving NF-κB-mediated EMT, suggesting a novel target for HCC treatment.

## INTRODUCTION

Worldwide, hepatocellular carcinoma (HCC) is one of the most common malignant tumors [1]. Although great progress has been made in the treatment of liver cancer in recent decades [2], the 5-year overall survival rate is still very low due to recurrence and metastasis [3]. Therefore, the discovery of the key genes and molecular mechanisms responsible for HCC progression will improve the outcomes of HCC patients.

Recently, epithelial-mesenchymal transition (EMT) plays an important role in tumorigenesis and development. In the EMT process, epithelial cells lose their epithelial properties and acquire a more aggressive (mesenchymal) phenotype, undergoing a series of substantially intricate biological and biochemical changes to promote motility and invasion [4]. Molecularly, EMT involves down-regulation of E-cadherin and up-regulation of N-cadherin. These changes in cell behavior and differentiation are regulated by key transcription factors and an intricate network of signaling pathways, including TGF-β, PI3K/Akt, Hippo, MAPK, and NF-κB [5–7].

Human RUFY3 (RUN and FYVE domain containing 3) is widely distributed in hippocampal neurons and can promote cone and axon growth [8]. Recently, RUFY3 also seems to play a crucial role in promoting cancer development. For example, RUFY3 predicts poor prognosis and promotes invasion and metastasis of lung adenocarcinoma by EMT [9]. Additionally, the interaction between RUFY3 and FOXK1 promotes invasion and metastasis of colorectal cancer (CRC) cells [10]. However, the role and potential mechanism of RUFY3 in the progression of HCC are still unclear.

In this study, we identified the expression level of RUFY3 in HCC tissues and the effect of RUFY3 on the progression of HCC patients. Additionally, we explored the mechanism responsible for the relationship between RUFY3 alterations and HCC cell behavior.

## RESULTS

### The expression of RUFY3 is up-regulated in HCC. The high expression of RUFY3 is associated with poor clinical prognosis of HCC patients

**Figure 1:**
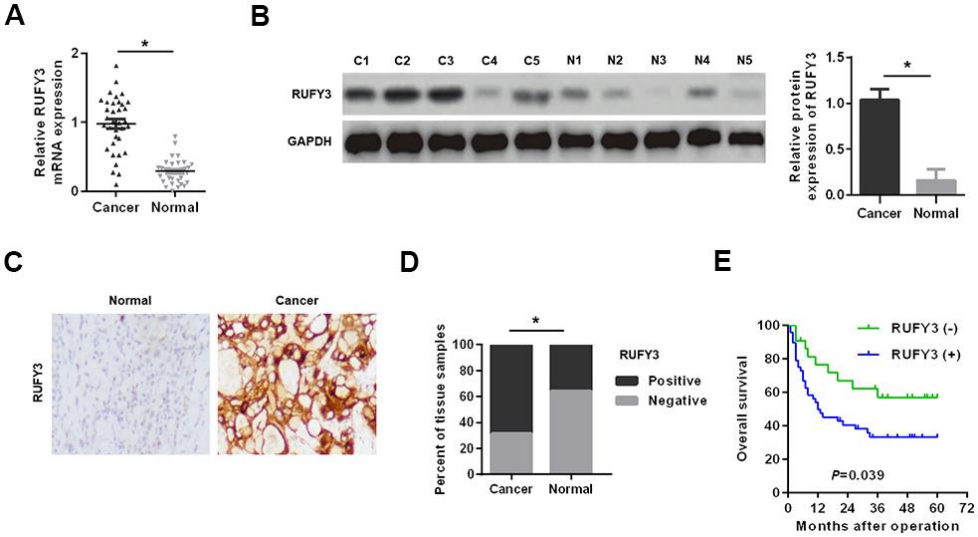
**Expression levels of RUFY3 in HCC tissues.** (**A**) Detection of RUFY3 mRNA expression in HCC tissues and adjacent normal tissues by qRT-PCR. (**B**) Detection of RUFY3 protein expression in HCC cancer tissues (C) and matched normal tissues (N) by western blot analysis. (**C**) Detection of RUFY3 expression in HCC tissues and matched normal tissues by IHC analysis. (**D**) Quantitative analysis of RUFY3 expression in HCC tissues and adjacent normal tissues according to IHC staining scores. (**E**) Kaplan-Meier analysis of overall survival for HCC patients with different levels of RUFY3 expression. *P<0.05.

As shown in Figure 1A, 1B, in 35 pairs of cases, the RUFY3 mRNA and protein levels in cancer tissues were significantly higher than those in adjacent normal tissues. Consistently, the IHC analysis of RUFY3 expression in all cases further demonstrated the similar outcome (Figure 1C, 1D).

Secondly, the correlation between the expression of RUFY3 and clinicopathological features of HCC patients showed that the high expression of rufy3 was significantly correlated with tumor size, microvascular invasion, TNM stage and Edmonson stage (*P* < 0.05) (Table 1). Kaplan Meier analysis showed that the overall survival of RUFY3 positive HCC patients was shorter than that of RUFY3 negative HCC patients (*P* = 0.039) (Figure 1E).

**Table 1:** Correlations between RUFY3 expression and clinicopathological features in patients with HCC.

**Features**	**n**	**RUFY3**		***P*-value**
		**Negative**	**Positive**	
Age (years)				
<60	51	17	34	0.574
≥60	19	5	14	
Gender				
Female	26	7	19	0.533
Male	44	15	29	
Tumor size (cm)				
<5	26	14	12	0.002*
≥5	44	8	36	
Tumor multiplicity				
Single	49	17	32	0.369
Multiple	21	5	16	
α-fetoprotein (ng/mL)				
<20	27	8	19	0.797
≥20	43	14	29	
HBV infection				
Negative	8	2	6	0.677
Positive	62	20	42	
Liver cirrhosis				
Absence	20	6	14	0.871
Presence	50	16	34	
Microvascular invasion				
Absence	28	4	24	0.012*
Presence	42	18	24	
TNM stage				
I + II	44	19	25	0.006*
III + IV	26	3	23	
Edmonson stage				
I + II	47	20	27	0.004*
III + IV	23	2	21	

RUFY3, RUN and FYVE domain containing 3; HCC, Hepatocellular Carcinoma.

### RUFY3 promotes EMT in HCC cells

**Figure 2:**
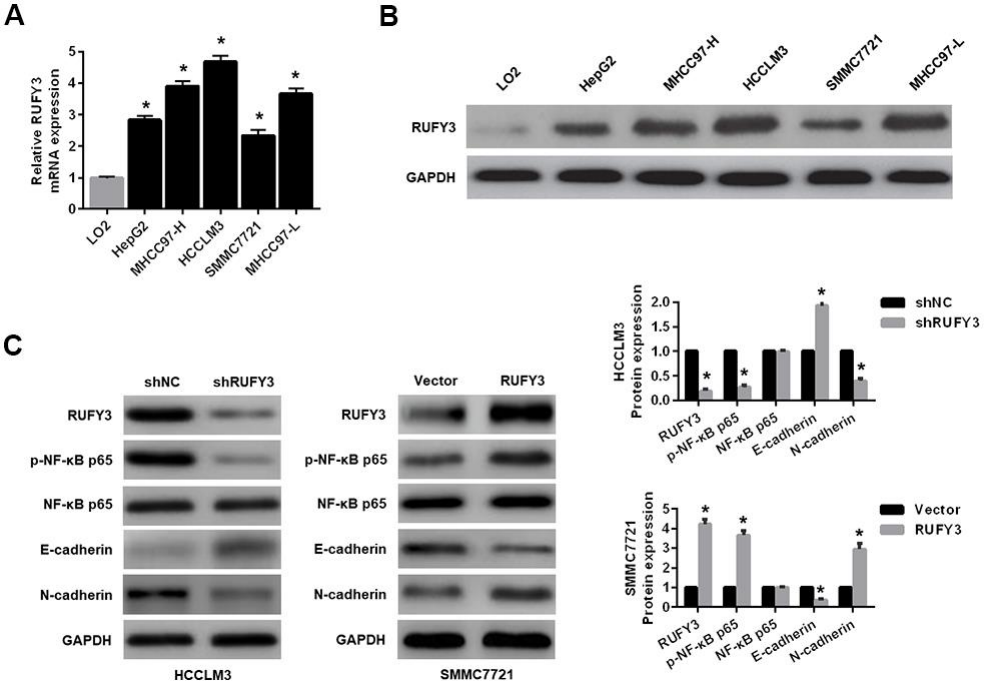
**Expression levels of RUFY3 in HCC cell and the effect of RUFY3 expression on NF-κB signaling-related markers.** (**A**) qRT-PCR detection of RUFY3 expression in five HCC cell lines and one normal liver cell line. (**B**) Western blot analysis of RUFY3 expression in five HCC cell lines and one normal liver cell line. (**C**) Western blot analysis of the effect of RUFY3 down-regulation or up-regulation on NF-κB signaling-related markers. *P<0.05.

As shown in Figure 2A, 2B, compared with normal human liver cell line (LO2), the mRNA and protein levels of RUFY3 in HCC cells were significantly increased. Among them, the expression levels of RUFY3 mRNA and protein in HCCLM3 cell were the highest. On the contrary, SMMC7721 cell showed the opposite outcome.

Second, regarding to the effect of RUFY3 alteration on EMT in HCC cells, we demonstrated that RUFY3 knockdown in HCCLM3 cell decreased the expression levels of p-NF-κB p65 and N-cadherin, and increased the expression level of E-cadherin. Conversely, RUFY3 overexpression in SMMC7721 cell led to the opposite outcome (Figure 2C).

### RUFY3 promotes the growth, migration and invasion of HCC cell *in vitro*

**Figure 3:**
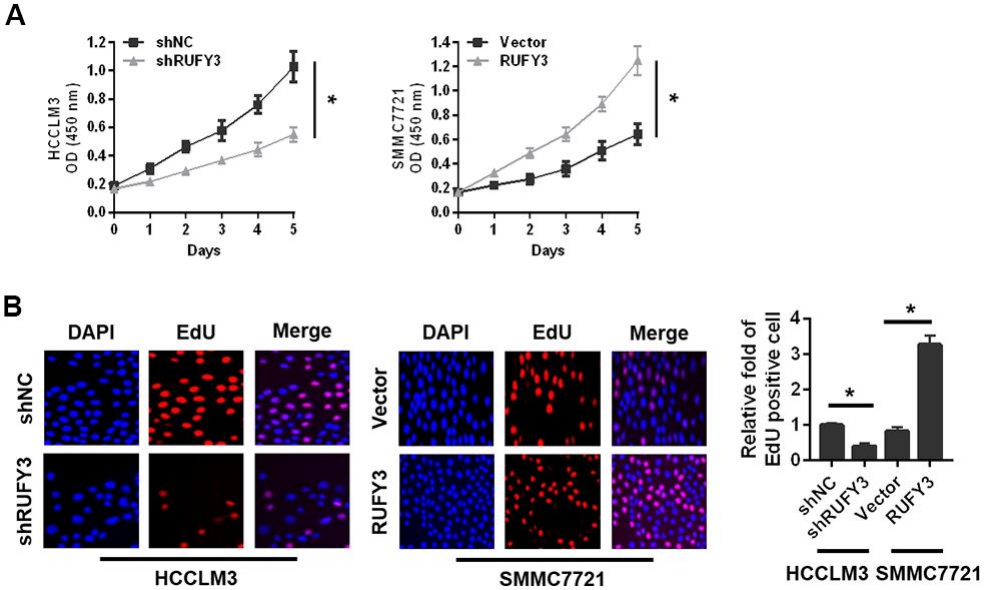
**Effect of RUFY3 down-regulation or up-regulation on HCC cell growth *in vitro*.** (**A**) Effect of RUFY3 down-regulation or up-regulation on HCC cell growth by CCK-8 assay. (**B**) Effect of RUFY3 down-regulation or up-regulation on HCC cell growth by EdU assay. *P<0.05.

As shown in Figure 3A, the proliferation of HCCLM3 cell transfected with shRUFY3 was observably lower than shNC cell. Conversely, RUFY3 overexpression in SMMC7721 cell resulted in the opposite outcomes (Figure 3A). Likewise, we further found that RUFY3 promoted the growth of HCC cell by EdU incorporation (Figure 3B).

**Figure 4:**
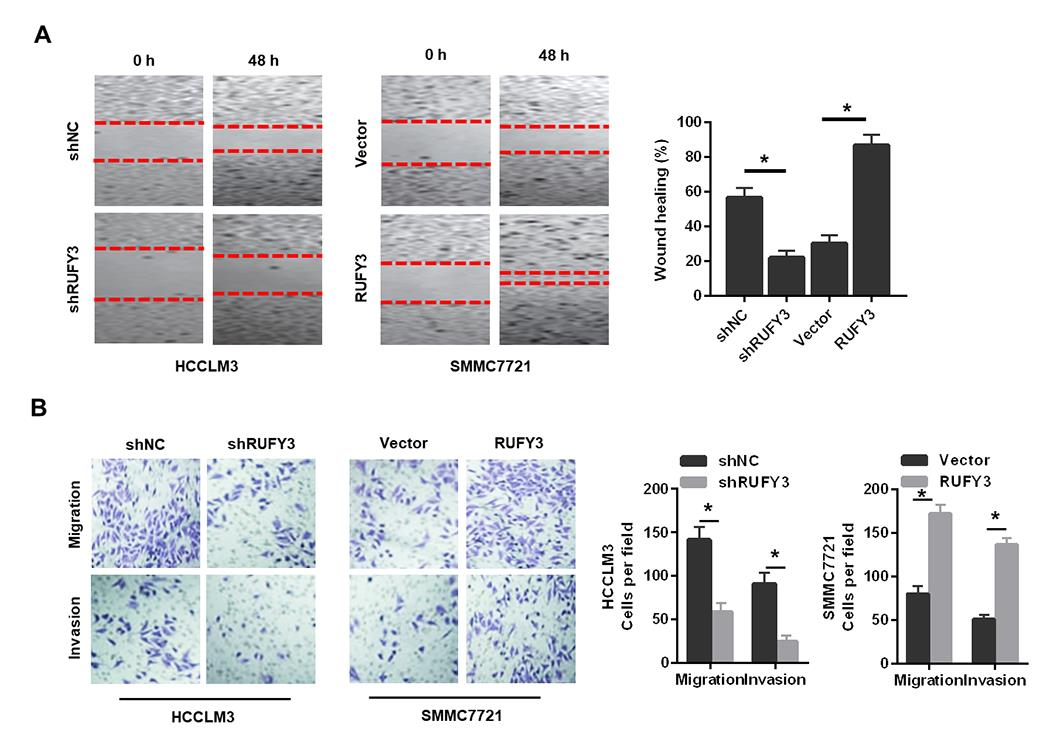
**Effect of RUFY3 down-regulation or up-regulation on HCC cell migration and invasion *in vitro*.** (**A**) Effect of RUFY3 down-regulation or up-regulation on HCC cell migration by wound-healing assay. (**B**) Effect of RUFY3 down-regulation or up-regulation on HCC cell migration and invasion by Transwell migration and invasion assays. *P<0.05.

Second, RUFY3 knockdown significantly reduced the wound healing ability of HCCLM3 cell (Figure 4A), whereas RUFY3 overexpression increased the wound healing ability of SMMC7721 cell. Additionally, transwell migration and invasion assay showed that the number of migration and invasion of HCCLM3 cell transfected with shRUFY3 was significantly lower than that of shNC cell. While the opposite outcomes were obtained from SMMC7721 cell, with significantly more cells passing through the Transwell membrane in the RUFY3 overexpression group than in the vector group.

In summary, these data indicated that RUFY3 promoted the growth, migration and invasion of HCC cell *in vitro*.

### RUFY3 promotes the growth and metastasis of HCC cell *in vivo*

**Figure 5:**
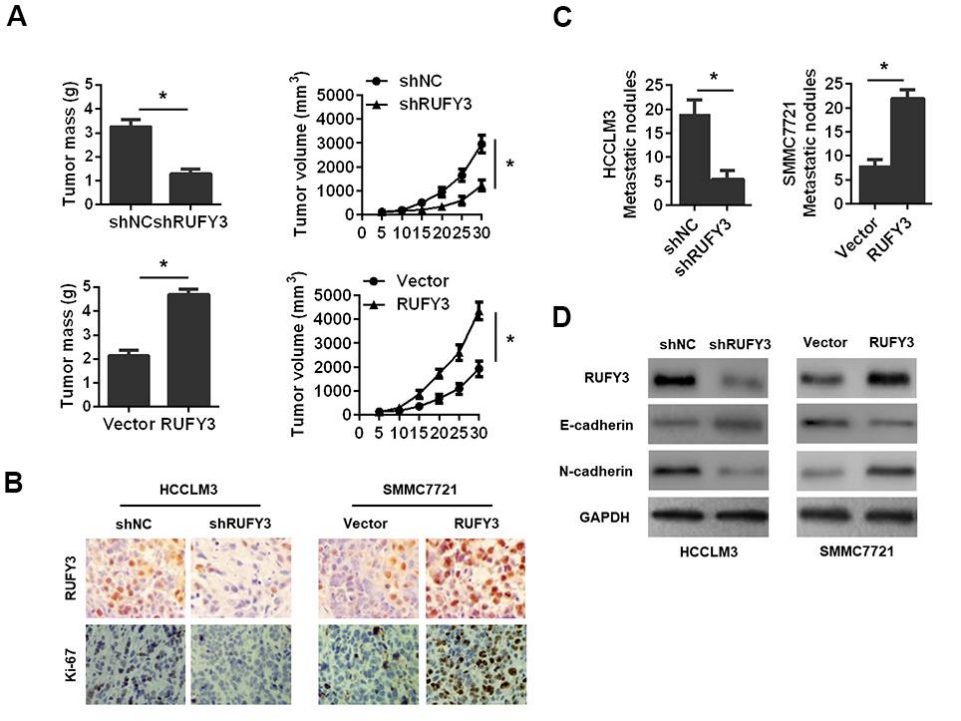
**Effect of RUFY3 down-regulation or up-regulation on HCC cell growth and metastasis *in vivo*.** (**A**) Effect of RUFY3 down-regulation or up-regulation on HCC cell subcutaneous xenograft growth in nude mice. Tumor mass and tumor volume are shown in the panels. (**B**) IHC analysis of RUFY3 and Ki-67 expression levels in xenograft tumor samples. (**C**) Effect of RUFY3 down-regulation or up-regulation on the metastasis of HCC cell *in vivo*. (**D**) Western blot analyses of the expression levels of RUFY3, E-cadherin, and N-cadherin in the lung metastatic nodules. *P<0.05.

We observed that RUFY3 knockdown in HCCLM3 cell markedly decreased while RUFY3 overexpression in SMMC7721 cell significantly increased tumor mass and tumor volume (Figure 5A). RUFY3 knockdown in HCCLM3 cell significantly decreased while RUFY3 overexpression in SMMC7721 cell significantly increased Ki-67 level (Figure 5B). Additionally, we found that RUFY3 knockdown markedly decreased the lung metastases of HCCLM3 cell *in vivo* (Figure 5C), and resulted in a noticeable reversal of EMT (Figure 5D). Conversely, RUFY3 overexpression in SMMC7721 cell led to the opposite results (Figure 5C, 5D). Collectively, these results indicated that RUFY3 promoted the growth and metastasis of HCC cell *in vivo*.

### RUFY3 activates NF-κB signaling in HCC

**Figure 6:**
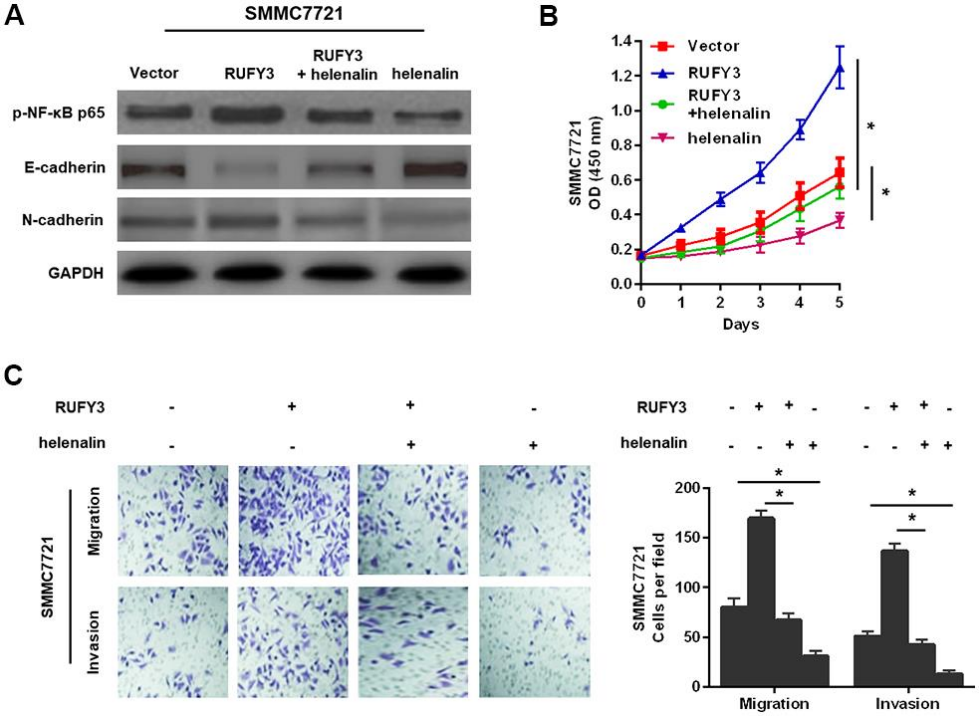
**NF-κB signaling involved in RUFY3-induced HCC cell EMT, growth, migration, and invasion.** (**A**) Western blot analysis of the levels of p-NF-κB p65, E-cadherin and N-cadherin in modified SMMC7721 cell treated with helenalin. (**B**) CCK-8 analysis of cell proliferation ability in modified SMMC7721 cell treated with helenalin. (**C**) Determination of cell migration and invasion abilities in modified SMMC7721 cell treated with helenalin. *P<0.05.

Given that the NF-κB signaling is involved in the growth, migration and invasion of various malignant tumor cells [11], we hypothesized that RUFY3 might promote these changes through activating NF-κB signaling pathway in HCC. To test the hypothesis, the specific NF-κB signaling inhibitor, helenalin [12], was used to perform studies more deeply involving modified HCC cells, based on the above-mentioned conclusion that RUFY3 promoted EMT in HCC cells. It was worth noting that the results of this study suggested that treatment with helenalin led to a significantly decreased expression of p-NF-κB p65 and a distinct reversal of RUFY3-induced EMT (Figure 6A) and markedly suppressed RUFY3-induced cell growth (Figure 6B), migration and invasion (Figure 6C). Collectively, these results indicated that NF-κB signaling-induced EMT was responsible for RUFY3-facilitated HCC cell growth, migration and invasion.

## DISCUSSION

HCC remains a very common and severe malignant tumor throughout the world. Although great progress has been made in treatment and research, tumor recurrence and metastasis are the main obstacles in the treatment of patients with HCC [13, 14]. Given that many gene changes are related to the growth, invasion and metastasis of cancer cell [15], it is very meaningful of searching for key molecular targets and the molecular mechanisms underlying HCC progression. It is worth noting that recent studies have shown that RUFY3 plays an important role in gene transcription regulation and promotes malignant biological behaviors of various cancers, such as cell growth and invasion [9, 10, 16]. For example, RUFY3 is located in the invasion foot rich in F-actin, which induces the formation of protrusion structure and plays an important role in the migration and invasion of gastric cancer cell. It is worth noting that blocking PAK1-RUFY3 signal transduction may be a potential strategy for the treatment of gastric cancer metastasis [16]. In CRC, RUFY3 is significantly up-regulated in cancer tissues compared with adjacent non cancer tissues, and plays a key role in cell invasion and metastasis [17]. Nevertheless, as far as we know, the special role of RUFY3 in the progression of HCC and the mechanism of RUFY3 in the occurrence and metastasis of HCC has not been clarified.

Herein, we found that the expression of RUFY3 in HCC was higher than that in adjacent non tumor tissues. Clinical analysis indicated that RUFY3 expression was positively associated with tumor size, microvascular invasion, TNM stage and Edmonson stage. Meanwhile, survival analysis showed that high expression of RUFY3 was associated with low OS in HCC patients, suggesting RUFY3 as a prognostic biomarker for HCC. Next, *in vitro* and *in vivo*, overexpression of RUFY3 promoted the growth, migration, invasion and metastasis of HCC cell; RUFY3 silence significantly reversed these events. Mechanistically, we found that RUFY3 promoted the HCC malignancy through the activation of EMT signaling. Thus, it is very meaningful of exploring the relationship between RUFY3 and EMT in HCC progression.

Recently, extensive evidence has confirmed that EMT is the basic process of disease progression in the stage of metastasis cascade, comprising of a series of intricate changes that cause a cell to lose its epithelial-like state and acquire a more mesenchymal-like phenotype [18].

During EMT, the transition from epithelial to mesenchymal states increases the migratory and invasive capabilities of individual cancer cell, and allows it to break away from its originating tissue and travel throughout the body [18]. Recently, it is interesting to note that the EMT pathway, as a novel therapeutic avenue for cancer treatment, could be targeted to prevent cell dissemination or to eradicate metastatic cell [18]. EMT is thought to be finely regulated by several intracellular and extracellular cues. Nevertheless, the major challenge remains regarding effective interventions focused on targeting various aspects of EMT to prevent cancer dissemination.

NF-κB is one of the most studied transcription factors. It was first found in the nucleus of B lymphocytes nearly 30 years ago. This protein plays a key role in regulating the human immune system. Its disorder is related to many chronic diseases, including asthma, cancer, diabetes, rheumatoid arthritis, inflammation and nervous system diseases [19]. Previous studies have confirmed that NF-κB played a key role in tumor progression and metastasis [20, 21]. In HCC, several oncogenic signal transduction pathways, such as MAPK, PI3K/AKT/mTOR, JAK/STAT, cMET, IGF and NF-κB, were found to play critical roles in tumor initiation and progression [22]. Mechanistically, the helenalin (an NF-κB inhibitor) [11] was employed to conduct studies involving modified HCC cells. The results showed that blocking NF-κB signal weakened the effect of RUFY3-promoting EMT, cell growth, migration and invasion. All in all, our data suggested that RUFY3 promoted the progression of HCC through NF-κB-induced EMT. However, the relationship between RUFY3 and NF-κB signal is worth further discussion. It is reported that the interaction between RUFY3 and FOXK1, as a new RUFY3 binding protein, promoted the invasion and metastasis of CRC. In addition, blocking PAK1-RUFY3 signal transduction may be a potential strategy for the treatment of gastric cancer metastasis. Therefore, it is important to evaluate whether RUFY3-induced EMT involves other molecules or some signaling pathways in the follow-up study.

In conclusion, this study shows that the high expression of RUFY3 is significantly associated with the clinical proliferation, metastasis and poor prognosis of HCC patients. In addition, this study confirms that RUFY3 can promote the growth, invasion and metastasis of HCC cell by activating NF-κB-induced EMT, which may provide a potential target for the treatment of HCC.

## MATERIALS AND METHODS

### Tissue samples

A total of 70 pairs of HCC and adjacent liver tissues were collected from patients undergoing hepatectomy in the First Affiliated Hospital of Soochow University. This study was approved by the ethics committee of the First Affiliated Hospital of Soochow University. All participants signed a written informed consent.

### Cell lines

Human hepatoma cell lines (HepG2, MHCC97-H, HCCLM3, SMMC7721 and mhcc97-l) and normal human liver cell lines (LO2) were obtained from ATCC. All cell lines were stored in DMEM (Thermo Fisher Scientific, USA) supplemented with 10% FBS (Thermo Fisher Scientific, USA) at 37° C and 5% CO_2_.

### Quantitative polymerase chain reaction (qRT-PCR)

Total RNA was extracted from liver cancer tissues or cells with Trizol reagent (USA), and then reverse transcribed into cDNA. The relative mRNA levels were monitored by SYBR Green Kit (Takara, China). The 2^-ΔΔCt^ method was used for quantitative analysis. The primer is summarized in Supplementary Table 1.

### Western blot assay

In short, after using RIPA buffer (Invitrogen, USA) to extract protein from cell or tissue lysates, the protein was isolated by SDS-PAGE and transferred to PVDF membrane (EMD-Millipore, USA). Then, after incubation with 5% skimmed milk, the membrane was incubated with primary antibody at 4° C. The cells were incubated overnight and then incubated with secondary antibody for 1 h. Finally, the enhanced chemiluminescence kit was used to image the membrane. Antibodies against RUFY3 and GAPDH were purchased from Abcam (UK). Antibodies against p-NF-κB p65, NF-κB p65, E-cadherin and N-cadherin were obtained from cell signaling technology (USA).

### Immunohistochemistry (IHC) and evaluation

Briefly, after deparaffinization and antigen retrieval, the samples were inactivated with 3% hydrogen peroxide, blocked with 5% BSA, incubated with primary antibody overnight at 4° C, and then incubated with secondary antibody (Beyotime, China) and Diaminobenzidine solution. Finally, the samples were imaged using a microscope.

Two pathologists, blinded to the data, evaluated the IHC staining results independently by the formula: IHC staining scores = the staining intensity (0, negative; 1, weak; 2, moderate; 3, strong) + the staining area (0, 0-5%; 1, 6-25%; 2, 26-50%; 3, >50%). Sum scores of <3 points or ≥3 points were regarded as negative or positive expression.

### Lentivirus infection

According to the manufacturer's instructions, the cells were transfected by Lipofectamine 2000 (Invitrogen, USA). Commercial short hairpin RNA (shRNA) and overexpression constructs of rufy3 were purchased from Genechem (Shanghai, China). Cells transfected with control shRNA or vector were used as control.

### Cell counting kit-8 (CCK-8) assay

Cells were seeded on 96 well plates and cultured for 0, 1, 2, 3, 4 and 5 days respectively. CCK-8 solution was used to measure the absorbance at 450nm to calculate the cell growth rate.

### EdU assay

In short, cells were seeded into 96 well plates. Cells were incubated with EdU reagent for 2 h, fixed with 4% paraformaldehyde, permeated with 0.2% TritonX-100, and stained with 4′, 6-diamino-2-phenylindole solution for 30 min. Cell viability was determined under fluorescence microscope.

### Wound healing assay

In short, cells were seeded in a 6-well plate. When the cells were fused, a sterile pipette was used to form the wound space. The wounds were imaged at designated time points (0 and 48 hours after the wound). Wound healing rate = (0 h width – 48 h width) / 0 h width × 100%.

### Cell migration and invasion assays

In short, the transwell chamber (8 μm pore size; Corning, USA) were coated with or not coated with Matrigel™ (BD Biosciences, USA) was used to measure cell invasion and migration. The cells were maintained in serum-free medium and inoculated in the upper cavity. 20% FBS was added to the medium as a chemical attractant. After incubation for 24 h, the cells on the bottom of the insert were fixed and stained. Finally, the migration and invasion cells were counted in five randomly selected regions by microscope.

### *In vivo* tumorigenesis and metastasis assays

All procedures were approved by the ethics committee of the First Affiliated Hospital of Soochow University. In order to investigate the effect of RUFY3 on tumor formation, cells were subcutaneously injected into nude mice, and then the tumor volume was measured with the following formula every five days: volume = (short diameter)^2^ × (long diameter) × 0.5. The nude mice were killed 30 days after injection, and the tumor was isolated for IHC analysis of RUFY3 and Ki-67 levels. In order to study the effect of RUFY3 on tumor metastasis, we injected cells into nude mice via tail vein. After 30 days, the lung tissues of nude mice were resected to count the metastatic nodules, and the expression levels of RUFY3, E-cadherin and N-cadherin were analyzed by Western blot.

### Statistical analysis

SPSS 21.0 was used for all statistical analysis. The data were expressed as mean ± standard deviation (SD) and analyzed by Student’s *t*-test. Chi-square test was used to compare the clinicopathological parameters. Kaplan-Meier method and log-rank test were used for survival analysis. *P* < 0.05 was considered significant.

## Supplementary Material

Supplementary Table 1
