# Supplementary material for: RUFY3 promotes the progression of hepatocellular carcinoma through activating NF-κB-mediated epithelial-mesenchymal transition
Source: Aging (Albany NY). 2021 Sep 12;13(17):21283–93. doi: 10.18632/aging.203444 (PMC8457573; doi:10.18632/aging.203444)
Supplement: Supplementary Table 1 [file aging-13-203444-s001.pdf]

SUPPLEMENTARY TABLE

Supplementary Table 1. Primers designed for qRT-PCR.

Genes	Sequence (5'-3')	
	Forward	Reverse
**RUFY3**	TTGTGGTGATGGAGCACTGT	GCCAGGCTCTTCCTCTACCT
**GAPDH**	AGGGGCCATCCACAGTCTTC	AGAAGGCTGGGGCTCATTTG
